# Aseptic Splenic Abscess as Precursory Extraintestinal Manifestation of Inflammatory Bowel Disease

**DOI:** 10.1155/2014/684231

**Published:** 2014-09-07

**Authors:** Joel Brooks, Gisoo Ghaffari

**Affiliations:** ^1^Heart of Lancaster Regional Medical Center, Department of Medical Education, 1500 Highlands Drive, Lititz, PA 17543, USA; ^2^Penn State Hershey Medical Center, Department of Medicine, 500 University Drive, Hershey, PA 17033, USA

## Abstract

Splenic abscesses are most often secondary to aerobic bacterial infections due to *Streptococcus*, *Staphylococcus*, and *Enterococcus* species of organisms. Sterile splenic abscesses rarely occur and diagnosis and treatment of those are challenging. We report a case of a previously healthy young female presenting with aseptic splenic abscesses as the initial manifestation of Crohn's disease along with a review of the literature on aseptic splenic abscess as an extraintestinal manifestation of Crohn's disease.

## 1. Introduction

Splenic abscesses are rare occurrences and are most commonly caused by bacterial infections with an incidence between 0.14 and 0.7% [1, 2]. There are fewer than 800 cases reported in the literature regarding splenic abscess. Aseptic splenic abscesses are much less common, with the majority of cases reported from Europe [3–5]. The majority of splenic abscesses are infectious; blood and aspirated fluid cultures often reveal the causative pathogen [6–8]. Aerobic bacteria,* Streptococcus*,* Staphylococcus*,* Enterococcus*, in addition to* Escherichia coli* (anaerobic) species, have been the causative organisms in most reports [9–12].

This report describes a young female with recurrent aseptic pustular skin lesions as well as sterile splenic abscesses refractory to antibiotic treatments. The small and large bowel biopsies as well as serology suggested Crohn's disease. In addition, the clinical, laboratory, and radiographic responses to prednisone and azathioprine offered additional support for the diagnosis of an inflammatory bowel disease (IBD).

## 2. Case Presentation

A 19-year-old Caucasian female residing in Pennsylvania presented to her primary care provider with pustular lesions on her right ankle. It was suspected that the patient developed cellulitis following a minor blunt trauma to the area. The skin lesions progressed despite outpatient treatment with clindamycin, requiring hospitalization. Significant laboratory findings included the following: elevated white blood cells (WBC; 15.1 cells/microL), erythrocyte sedimentation rate (ESR; 80), and C-reactive protein (CRP; 7.6). Wound and blood cultures along with a right ankle joint aspiration were negative, as well as magnetic resonance imaging (MRI) of the bone and indium-labeled WBC scan for osteomyelitis. The antibiotic was changed to intravenous vancomycin followed by oral clindamycin plus trimethoprim-sulfamethoxazole for outpatient treatment. Two weeks after completion of this therapy, the patient presented with similar lesions on the dorsum of the left foot. Bone MRI showed microabscesses that were incised yielding* E. coli* from a single culture. She was subsequently treated with 5 weeks of ciprofloxacin and vancomycin for* E. coli*/Methicillin Resistant* Staphylococcus aureus* (MRSA) infection.

**Figure 1:**
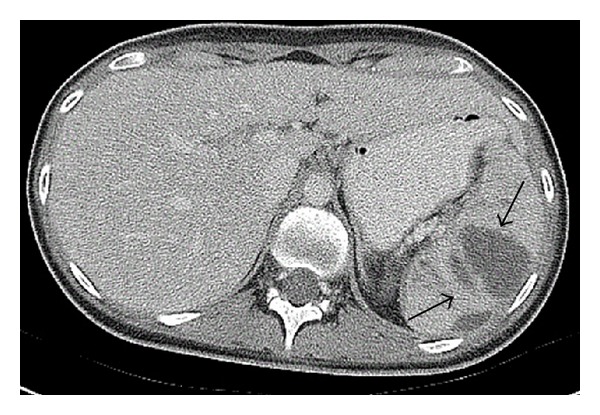
Splenic abscesses noted on CT scan (arrows).

Three weeks after completing antibiotic treatment, she presented with abdominal pain that was attributed to a urinary tract infection. When the pain did not resolve with empiric antibiotic treatment, an abdominal CT scan was performed which revealed multiple low-density splenic lesions, the largest of which measured 4 × 2.9 cm (Figure 1). The antibiotic was changed to intravenous ceftriaxone, followed by daptomycin, ciprofloxacin, and metronidazole. A repeat abdominal CT one month later showed increased size and number of the splenic lesions and a small intra-abdominal fluid collection near the cecum. During this time, all aerobic, anaerobic, mycobacterial, and fungal blood cultures remained negative as well as a transesophageal echocardiogram for endocarditis. The CT-guided aspiration of the abdominal fluid collection was sent for cultures and cytology and returned negative for any infectious or malignant process.

**Figure 2:**
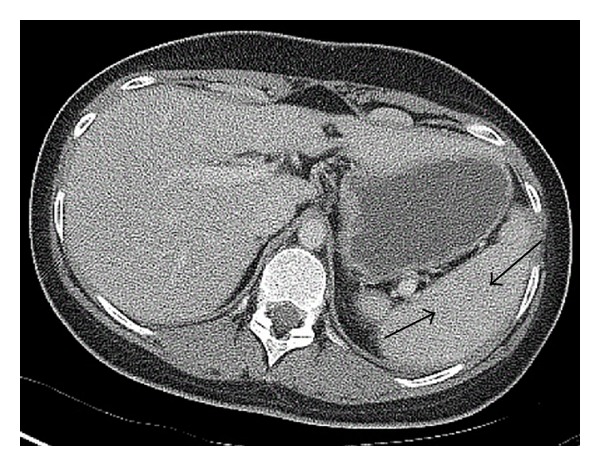
Resolution of the patient's splenic abscesses 6 months following treatment initiation (previously effected regions denoted by arrows).

Ultimately, all antibiotics were discontinued due to side effects and lack of clinical improvement. The extensive workup included serologic testing for infectious processes, skin biopsies, thorough rheumatologic tests, and comprehensive immunologic evaluation for primary immune deficiencies including chronic granulomatous diseases and secondary immune deficiencies as well as an upper endoscopy which was unrevealing. A colonoscopy with biopsy revealed regions of focal mild acute ileitis with villous blunting and fibrosis. The right colon demonstrated mildly increased lymphoplasmacytic infiltration in the lamina propria. The left colon and rectum displayed acute cryptitis. These findings indicated acute colitis and proctitis consistent with Crohn's disease. An elevated IgA and IgG anti-Saccharomyces cerevisiae antibodies (ASCA) and negative perinuclear antineutrophil cytoplasmic antibodies (p-ANCA) further supported this diagnosis. The patient was started on oral corticosteroids and demonstrated symptomatic improvement during her hospital stay with eventual resolution of splenic lesions within 6 months (Figure 2). She was followed closely by gastroenterology within the first few months of her diagnosis and was transitioned from prednisone to azathioprine with continued resolution of both skin and splenic findings with no further evolution of symptoms. A repeat colonoscopy performed 8 months after the initial diagnosis was normal. The patient continues to be followed by gastroenterology annually.

## 3. Discussion

This report describes a young female with recurrent aseptic pustular skin lesions as well as sterile splenic abscesses refractory to antibiotic treatments. The results of the small and large bowel biopsies as well as serology suggested Crohn's disease. The responses to prednisone and azathioprine offered additional support for the diagnosis of IBD.

PubMed and OVID were searched for the current English literature using the following keywords: splenic abscess, IBD, Crohn's disease, and aseptic splenic abscess. The search revealed case reports and case series with a variable number of patients, ranging from 1 to 287 [1, 6, 13–20]. Splenic abscesses are uncommon and, based on autopsy reviews, the incidence between 0.14 and 0.7% has been reported [1, 2]. Aseptic splenic abscesses are rare, with the majority of cases being reported by European countries [3–5].

The signs and symptoms of splenic abscess are nonspecific and may include fever, abdominal pain, and leukocytosis [9, 17, 21, 22]. The causative organisms in most reports are aerobic bacteria such as* Streptococcus*,* Staphylococcus*, and* Enterococcus* or anaerobic bacteria such as* Escherichia coli* [9–12]. A group in Spain identified* Mycobacterium tuberculosis* (M-TB) as the causative pathogen in 36.4% of 22 cases [11]. Two groups from Japan and one group from Taiwan noted that gram-negative bacillus accounted for 46.7%–60% of their cases [6, 23]. Predisposing factors, such as diabetes mellitus, malignancies, immunosuppressive medications, or immunodeficiency disorders, often play a role in the development of splenic abscess and should be considered when a splenic abscess is found [8, 21, 22, 24–26].

Few case series have specifically looked at patients with sterile splenic abscesses. One case series found M-TB to cause culture negative splenic abscesses. The case series mentioned above reported that approximately 13% of patients have had sterile splenic abscesses [27]. A report from France outlined the clinical course in 30 patients. The mean age at diagnosis was 29 years and main clinical manifestations were fever, abdominal pain, and weight loss. Twenty-two of these patients had leukocytosis and twenty-one had IBD diagnosed by colonoscopy and/or barium swallow. Serological studies with elevated CRP > 6, antinuclear antibodies, and rheumatoid factor were found in this group. Tests for ANCA and anti-Saccharomyces cerevisiae antibodies (ASCA) were performed. Of the 21 patients diagnosed with IBD, the diagnosis preceded the splenic abscesses in 7 cases, was identified at the same time as the abscesses in 7 cases, and appeared secondarily in 7 cases [13]. A recent report published discussed a case of a 15-year-old girl who developed multiple aseptic abscesses. She was treated with a combination of metronidazole, meropenem, and prednisone 2 mg/kg therapy with symptom resolution. The subject was subsequently found to have autoimmune thyroiditis [28].

There are scarce reports listing splenic abscesses as the first sign of Crohn's disease. A recent case report in 2010 describes a patient with aseptic splenic abscesses as the first manifestation of Crohn's disease. Unlike our case, that patient was male and in his 60s. He experienced recurring splenic abscesses unresponsive to antibiotic therapy and had a negative workup for infections, malignancies, lymphoproliferative processes, and immunodeficiencies. Crohn's disease was eventually diagnosed after an enteroscopy with biopsy demonstrated cryptitis, inflammatory cellularity in the lamina propria, and epithelioid granuloma consistent with Crohn's disease [20].

## 4. Conclusion

The presentation of pustular skin lesions, splenic abscesses, leukocytosis, elevated ESR, and CRP suggested an infectious etiology in our patient. After an extensive negative workup for infectious etiologies and a thorough immunological evaluation in addition to failure to respond to antibiotic therapy, an alternative diagnosis was suspected. The results of the colonoscopy with biopsy and serology were consistent with the diagnosis of Crohn's disease. While this condition has been documented internationally, there has been little consideration of IBD on the differential diagnosis for patients with aseptic splenic abscesses in the United States. Colonoscopies with biopsies are needed for diagnosis. The institution of immunosuppressive medications can lead to symptomatic improvement and resolution of splenic lesions that are due to IBD [11, 20, 21, 29].
